# Characterization and cytocompatibility of 3D porous biomimetic scaffold derived from rabbit nucleus pulposus tissue in vitro

**DOI:** 10.1007/s10856-020-06480-9

**Published:** 2021-01-20

**Authors:** Yu Zhang, Wei Tan, Mingxin Wu, Jin Sun, Wei Cao, Chu-Song Zhou, You Wu

**Affiliations:** 1 Department of Orthopaedics, General Hospital of Southern Theatre Command of PLA, Guangzhou, 510010 Guangdong, PR China; 2 Department of Spinal Orthopedics, Huizhou Third People’s Hospital, Guangzhou Medical University, Huizhou, 516002 Guangdong PR China; 3 Department of Spine Surgery, The Third Xiangya Hospital of Central South University, Changsha, 410013 Hunan, PR China; 4 Department of Spinal Orthopedics, The first people’s hospital of Huaihua, Huaihua, 418000 Hunan PR China; 5 Department of Orthopaedics, Zhu-Jiang Hospital of Southern Medical University (First Military Medical University), Guangzhou, 510282 Guangdong, PR China

## Abstract

Intervertebral disc (IVD) degeneration is one of the most important causes of lower back pain. Tissue engineering provides a new method for the experimental treatment of degenerative disc diseases. This study aims to develop a natural, acellular, 3D interconnected porous scaffold derived from the extracellular matrix (ECM) of nucleus pulposus. The nucleus pulposus (NP) was decellularized by sequential detergent-nuclease methods, including physical crushing, freeze-drying and cross-linking. These 3D porous scaffolds were fabricated with a high porosity of (81.28 ± 4.10)%, an ideal pore size with appropriate mechanical properties. Rabbit bone marrow mesenchymal stem cells (rBMSCs) were seeded and cultured on the scaffolds. And the mechanical tests showed the compressive elastic modulus of the scaffolds cultured for 4 weeks reached 0.12 MPa, which was better than that of the scaffolds cultured for 2 weeks (0.07 MPa) and that of the control group (0.04 MPa). Scanning electron microscopy (SEM), histological assays, molecular biology assays revealed that the scaffolds could provide an appropriate microstructure and environment for the adhesion, proliferation, migration and secretion of seeded cells in vitro. As assays like histology, immunohistochemistry and the real-time qRT-PCR showed, NP-like tissues were preliminarily formed. In conclusion, the 3D porous scaffold derived from NP ECM is a potential biomaterial for the regeneration of NP tissues.

**Figure:**
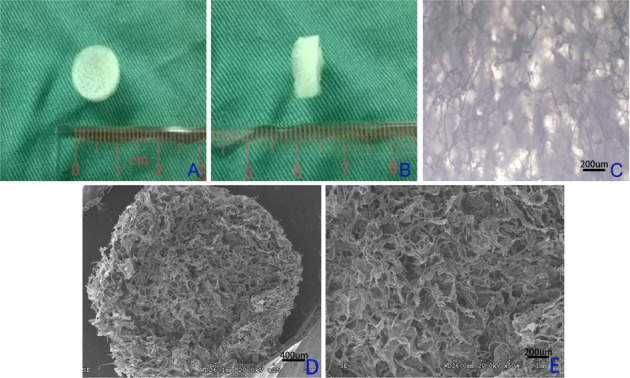
A natural, acellular, 3D interconnected porous scaffold derived from the extracellular matrix (ECM) of nucleus pulposus was developed by sequential detergent-nuclease and freeze-drying method, which can reduce the damage of protein activity to the minimum. It is very similar to the composition and internal environment of the natural nucleus pulposus, because it derived from the natural nucleus pulposus. Scanning electron microscopy (SEM), histological assays, molecular biology assays revealed that the scaffolds could provide an appropriate microstructure and environment for the adhesion, proliferation, migration, and secretion of seeded cells in vitro.

A natural, acellular, 3D interconnected porous scaffold derived from the extracellular matrix (ECM) of nucleus pulposus was developed by sequential detergent-nuclease and freeze-drying method, which can reduce the damage of protein activity to the minimum. It is very similar to the composition and internal environment of the natural nucleus pulposus, because it derived from the natural nucleus pulposus. Scanning electron microscopy (SEM), histological assays, molecular biology assays revealed that the scaffolds could provide an appropriate microstructure and environment for the adhesion, proliferation, migration, and secretion of seeded cells in vitro.

## Introduction

Intervertebral disc degeneration (IDD) can result in lower back pain and significant socioeconomic burden. The regeneration potential of human intervertebral disc is limited. Generally, the exact aetiology of IDD remains uncertain. However, it is well known that IDD is a multifactor process, including genetic, biochemical, biomechanical factors, etc [1–3], which can result in the changes of cell-conditioned medium [4] within the extracellular matrix (ECM) of intervertebral disc (IVD). IVD is composed of nucleus pulposus (NP) and annulus fibrosus. IVD degeneration is commonly considered to result from NP degeneration [5, 6]. NP is a gel-like substance in the central region of an IVD, containing a large number of aggrecan and collagen II [2, 7]. The major reason of NP degeneration is the decrease in the number and vitality of NP cells, which results in a series of problems, such as the decline of aggrecan, collagen II, and water content of NP tissue, as well as the reduction of buffering capacity of NP. All of the above events result in NP collapse and IVD height loss. Currently, there is no effective method to prevent its occurrence and development, and no ideal measure to treat IDD itself. Indeed, the traditional clinical treatment of IDD, which includes conservative treatment (drug and physical therapy) [8] and surgery (spine fusion and total disc removal) [9], is not absolutely avoided from concerns about possible comorbidities, cost-effectiveness, secondary risks and long-lasting outcomes. Meanwhile, these therapies can only alleviate the symptoms of lower back pain, but fail to treat the underlying cause of degeneration. Surgical treatment cannot reverse the degeneration of IVD, and may even accelerate it. Due to the great clinical need for effective methods to enhance or directly repair NP, NP repair is a subject worthy of intensive investigation in orthopedic reconstruction. Interestingly, can a new biomaterial be found as a substitution for the NP tissue?

The development of tissue engineering and regenerative therapeutic strategies has brought new hope for the repair and regeneration of degenerated IVD. These strategies have been developed mainly for the repair and regeneration of NP of the degenerated IVD [10–12]. Over the past few years, great efforts have been devoted to the design of innovative biomaterials for the repair/regeneration of nucleus pulposus in laboratory settings, such as bovine biomimetic NP scaffold [13], multi-polymers [14], injectable hydrogels [15–18], and cellular and cell-laden hydrogels [19, 20]. However, scaffold-based NP repair without giving much regard to NP could not recover the complex microenvironment structure for cell attachment, proliferation, and migration, which might make the disc degenerative change continuously exacerbate. Since it is very difficult to completely imitate the complicated natural composition and microenvironment of NP, the selection of scaffold-based materials and optimization of scaffold architecture are critical for NP tissue engineering. In addition, the processes of cell attachment, proliferation and migration in the regenerated NP are strongly influenced by the composition and structure of ECM, which is extremely similar to that of normal NP [21].

Therefore, it is generally believed that NP ECM-derived 3D porous scaffolds would be effectively utilized to support the regeneration of NP. Moreover, research has demonstrated that NP ECM-derived 3D porous scaffolds have many significant advantages, such as good biocompatibility, proper mechanical properties, low immunogenicity, and appropriate degradation rates. Currently, ECM scaffolds have been widely applied in clinical settings, and produced into various new tissues, such as bladder [22], vascular [23], genuine leather [24], heart valve [25], and cartilage tissues [26], have been successfully developed. However, the relationship between the extent of decellularization (the porosity and pore size distribution) of NP ECM scaffolds and their in vitro cytocompatibility of the rabbits has rarely been reported. Due to its superior construction in the microenvironment, NP ECM scaffold proves to work effectively and further enables in vivo the cell proliferation of the nude mice. Nevertheless, there is little research concerning the use of NP ECM scaffolds, except for the reports by Mercuri [27] which apply a combination of chemical detergents and nuclease treatment to the complete decellularization of porcine NP. However, the NP ECM scaffolds adopted by Mercuri and his colleagues were with small pore sizes. Jung [26] reported the development of 3D porous cartilage acellular matrix scaffolds fabricated with the simple freeze-drying method. Because of the great similarity in the biochemical composition, biomechanical function, and cellular type between NP and cartilage tissue, this research attempted to fabricate novel NP ECM-derived porous scaffolds with interconnected pores for NP TE by combining the methods of Mercuri and Jung CS.

In this study, NP ECM-derived scaffolds were obtained by adopting chemical detergents, nucleases, and freeze-drying methods. The morphology and surface composition of the scaffolds were studied with the help of optical microscopy and scanning electron microscopy (SEM). The cytocompatibility of scaffolds, such as cell morphology and cell proliferation, was investigated with SEM, DAPI 4,6-diamino-2-phenyl indole (DAPI), and MTT (3-(4,5-dimethylthiazol-2-yl)-2,5-diphenyl tetrazolium bromide) assays. Furthermore, histopathological and immunohistochemical analyses were conducted. The objective of this study is to provide an effective 3D porous ECM-derived scaffold as the substitution for the natural NP by means of biomimetic decellularization.

## Materials and methods

### Materials

All chemicals were purchased from Sigma-Aldrich Corporation, and goat anti-rabbit collagen II monoclonal antibodies (CP18.100 ug) from Millipore. All the reagents were of analytical grade and were used without further purification.

### Scaffold preparation

**Fig. 1:**
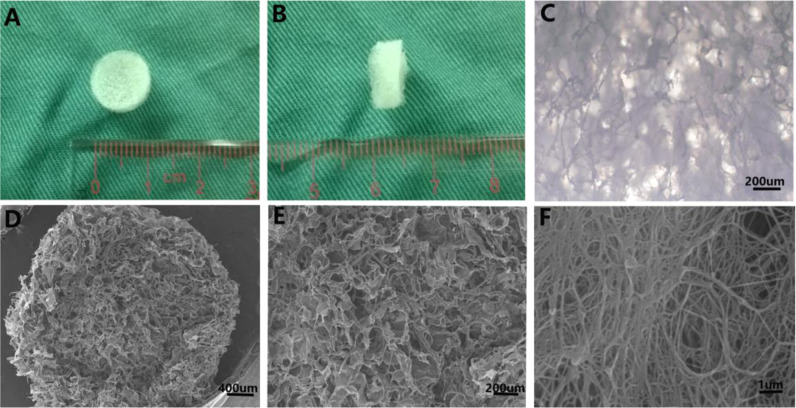
**a**, **b** Macroscopic images of NP ECM-derived 3D porous scaffolds, which represented gross appearance of scaffolds. These scaffolds had a diameter of 10 mm and a height of 3 mm. **c** Optical microscopy images of scaffolds. **d**, **e** SEM micrograph of scaffolds at different magnifications, box D represents 250× and box E represents ×500. **f** SEM micrograph of native nucleus pulposus at ×10,000

NP samples were obtained from T10 to L7 levels of the thoracolumbar segments of 60 white New Zealand adult rabbits (2.3–3.3 kg). Bone chips and excess blood were removed from each sample by repeated washing with phosphate buffer solution (PBS) (Sigma, St. Louis, MO, USA). After the removal of fiber ring and soft tissue with a blade, the NP tissues were carefully collected. These NP samples were then soaked in sterile PBS at 4 °C. After the immersion in PBS, the NP samples swelled dramatically. The NP samples were placed into 250 ml conical flasks containing 200 ml ice-cold decellularization solution (DS). Based on the report by Mercuri [27], we optimized the decellularization procedure by altering the vibration speed, time-course, and concentration. According to the detailed protocol given in Ref. [27], the DS was prepared in 50-mM Tris (hydroxymethyl-aminomethane) buffer (pH 7.5), which contains 0.2% (w/v) ethylenediamine tetraacetic acid, 0.3% (V/V) TritonX-100, 0.5% (V/V) sodium deoxycholic acid, and 1% (g/100 ml) phenylmethanesulfonyl fluoride. The NP samples were stirred on an orbital shaker at the rate of 120 rpm at room temperature for 12 h. The samples were then removed from the DS and carefully rinsed with 70% ethanol and distilled water several times. These steps were repeated three times, respectively. The samples were incubated in the 100 mL nuclease mixture (750 mU/mL deoxyribonuclease and 750 mU/mL ribonuclease) in PBS (pH 7.4) at 37 °C for 30 min, and thoroughly rinsed with deionized water for 30 min while shaking at 120 rpm. Subsequently, some decellularized NP samples were obtained and retained for the determination procedure. Thereafter, the other samples were stirred in deionized water for at least 30 min with an electric homogenator machine (JB50-S, Shanghai, China). The homogenized liquid was completely transferred to a centrifuge tube, and the sample was centrifuged for 5 min at 12000 r/min. The upper liquid layer was poured out, and the precipitate was frozen at −70 °C and freeze-dried to obtain the porous NP matrix microfilament. The dry weight of the NP matrix microfilament was measured, and the microfilament was mixed with a suitable amount of 2.5% (w/v) deionized water. About 1000 µl of 2.5% (w/v) suspension was added to a Teflon mould (a cylindrical vial with a diameter of 15 mm and a depth of 20 mm), which was then frozen at −20 °C for 2 h and −80 °C for 1 h, respectively, in a vacuum freeze dryer (LGJ-12, Beijing, China), so as to obtain the 3D porous NP ECM-derived scaffolds (as shown in Fig. 1a, b). The scaffolds were finally sterilized by ^60^Co γ-irradiation at a dose of 25 kGy for all tests.

### Physicochemical characterization

#### Basic characterization

A rough observation of the color, shape, and size of the scaffolds was carried out. The scaffold specimens were cut into 1 mm thick in size. Optical microscopy (Olympus microscope, Japan) and SEM (Quanta-400, Fei, Netherlands) were applied to study the cross-sectional morphology of the samples. These samples were sputter-coated with a layer of gold about 10 nm thick for SEM observations at 20 kV. The average pore size was determined based on the measurement of 50 random pores from the SEM images.

#### Swelling properties

The scaffolds had been immersed in distilled water at room temperature for 24 h. After the removal of excess water, the wet weight of the scaffold (*W*_s_) was determined. Samples were then dried in a vacuum −80 °C freeze dryer overnight and the dry weight of scaffolds (*W*_d_) was determined. The swelling ratio of the scaffold and the water content in the scaffold was calculated as follows:

$${\mathrm{Swelling}}\,{\mathrm{ratio}} \,=\, \left( {W_{\mathrm{s}} \,-\, W_{\mathrm{d}}} \right)/W_{\mathrm{d}} \,\times\, 100\% .$$

$${\mathrm{Water}}\,{\mathrm{uptake}}\,\left( \% \right) \,=\, \left( {W_{\mathrm{s}} \,-\, W_{\mathrm{d}}} \right)/W_{\mathrm{s}} \,\times\, 100\%$$

#### Porosity

The porosity of the scaffold was determined by the liquid displacement method. A calibrated test tube was selected, and a certain volume of anhydrous ethanol was added to the tube (recorded as V_1_). The scaffold was then placed in the tube and soaked for 5 min. The sample was repeatedly extruded until there was no overflow of bubbles, and the required volume was recorded as V_2_. The volume of the skeleton structure was the difference between V_2_ and V_1_. The scaffolds were then removed and impregnated with ethanol, and the residual ethanol volume was noted as V_3_. The pore volume of the scaffold was the difference between V_3_ and V_1_. The total volume was calculated using the following formula: Vtotal = (V_2_ − V_1_) + (V_1_ − V_3_) = V_2_ − V_3_. The porosity was calculated according to the formula: (V_1_ − V_3_)/(V_2_ − V_3_) × 100%. A total of six samples were tested, and each experiment was performed in triplicate.

#### MTT assay

MTT (Sigma, St. Louis, MO, USA) assay [28] was used to evaluate cellular energy metabolism and indicate cell proliferation indirectly. According to the Chinese national standard (GB-16886.5T-2003) for biological evaluation, the in vitro cytotoxicity experiment was performed at 37 °C with at least 24 h of scaffold extraction. Scaffold leaching solutions with different concentrations of 25, 50, and 100% were prepared and divided into four groups (*n* = 6), namely A, B, C, and D (single control). According to the previously reported protocol [29], rabbit bone marrow stem cells (rBMSCs) (Cyagen, USA) were applied to investigate the cytotoxicity of 3D porous NP ECM-derived scaffolds. The cells were cultured in the 25 cm^2^ tissue culture flasks at 37 °C in a humidified atmosphere of 5% CO_2_ in air. The applied cell culture medium was Dulbecco’s Modified Eagle Medium, i.e., nutrient mixture F12 (DMEM/F12, Gibco, USA) culture medium, which contains 8% fetal calf serum (FBS, Gibco, USA). The passage 3 (P3) generation of rBMSCs (Cyagen, USA) with a density of about 5 × 105 cells/cm^2^ was suspended in the culture medium, and then dispersed over the four groups in a 96 well plate by MTT assays. The assays were performed at the second, fourth, and sixth day, respectively.

#### Mechanical properties

To analyze the mechanical properties of the blank scaffolds, scaffolds cultured for 2 and 4 weeks, a compression test was performed at room temperature by means of a Zwick universal testing machine (Zwick Z010, Zwick GmbH, Germany) with a constant strain rate of 1 mm min^−1^. The samples were placed into 15 mL of simulated body fluid (SBF) and incubated at room temperature for 6 h. SBF solution (pH 7.25) was prepared according to the detailed protocol given in [30, 31]. Five samples were evaluated respectively in each group. The compressive stress and strain curves were graphed. The average compressive strength, compressive modulus and standard deviation were determined. The compressive modulus was defined by the slope of the initial linear section of the stress–strain curve.

### Cytocompatibility in vitro

#### Cell seeding experiments

According to the previously reported protocol [29], the cytocompatibility of NP ECM-derived porous scaffolds was studied with the passage 2 (P2) of rBMSCs (Cayagen, USA). The cells were cultured in the 25 cm^2^ tissue culture flasks at 37 °C in a humidified atmosphere of 5% CO_2_ in air. P5 of NP cells were obtained using the cell culture medium DMEM/F12 coupled with 4% FBS (Gibco, USA), 100 U/ml antibiotic–antimycotic (Gibco, USA), 40 µg/ml proline (Sigma, USA), 100 nmol/L dexamethasone (Sigma, USA), 0.1 mmol/L vitamin C (Sigma, USA), 100 U/ml antibiotic–antimycotic (Gibco, USA), 1% ITS^+premix^ (Cyagen, USA), and 20 ng/ml TGF-β_1_ (PeproTech, USA) for the cell seeding experiment. The cells with a density of about 2 × 107 cells/cm^2^ were suspended in the culture medium, and dispersed over the scaffolds in a 24 well plate, which were sterilized by ^60^Co γ-irradiation at a dose of 25 kGy.

#### Cell proliferation and morphology

The total DNA content of NP cells in the cell-seeded scaffolds was determined by fluorescence spectrophotometer (Thermo Fisher Scientific, USA). The assays were performed at day 4, week 2 and week 4, respectively. Cells cultured in wells without scaffold at a density of 2 × 107 cells/cm^2^ were served as controls. The cell-seeded scaffolds were washed with PBS (Sigma, St. Louis, MO, USA), crushed and air-dried under the laminar airflow for 10 min and frozen at −50 °C for 24 h. The scaffolds were then lysed with Nuelei Lysis (Promega, USA) solution for 30 min at room temperature. Afterwards, the lysed product was added to 100 μl DNA rehydration working reagent (A1125, Wizard genomic DNA purification kit, Promega, USA) and incubated at 37 °C. The absorbance of these samples was measured at 37 °C with a 458 nm fluorescence spectrophotometer. The six samples in each group were measured at each time point and the results were recorded in U L^–1^. Morphological changes of the cell-seeded scaffolds cultured in the induced medium were observed at every time point in vitro. The pure scaffold without seeded cells and the normal NP were used as the negative and positive control, respectively.

#### Cell viability and attachment

After 2 days of culture, the cells cultured on the scaffolds were investigated by a fluorescence microscope (Olympus, Japan), and the cell viability of adherent rBMSCs was assessed by the live/dead cell viability assay kit (Kergen Corporation, Nanjing, China). The cell nuclei were stained with live/dead staining solution (Calcein AM/EthD-1). Cells were incubated in 5 μg/ml Calcein AM/EthD-1 solution diluted with PBS buffer at 37 °C for 30 min. The samples were then washed with PBS buffer for three times. A mixture of 50% glycerin and 50% PBS buffer was added to keep the samples wet during the examining period. According to the emission wavelength of Calcein AM and EthD-1, the filter was set to 570 LP and the laser was set to 495 nm in the fluorescence mode. In the reflection mode, the laser was set to 495 nm while the filter was set to 515/635 nm, and the live cells were colored green while dead cells were stained red. The cell-seeded scaffold samples treated by pure alcohol were used as control. For SEM observations, the cell-seeded scaffolds were fixed with 2.5% glutaraldehyde at room temperature for 20 min, dehydrated through a series of graded alcohol solutions, and then freeze-dried overnight. All samples were coated with gold sputtering before SEM examinations.

#### Cell labeling assay

The cell labeling assays [32] were performed at week 2 and week 4, respectively. Cells cultured on the scaffolds were observed with a fluorescence microscope (Olympus, Japan) after being washed with PBS twice. The cell nuclei were colored with DAPI (Sigma, St.Louis, MO, USA) and the cell membrane were colored with 3H-Indolium, 5-[[[4-(chloromethyl)benzoyl]amino]methyl]-2-[3-(1,3-dihydro-3,3-dimethyl-1-octadecyl-2H-indol-2-ylidene)-1-propenyl]-3,3-dimethyl-1-octadecyl-, chloride (CellTracker^TM^ CM-DiL, Invitrogen, USA). Cells were incubated in 5 μg/ml CM-DiL solution diluted with PBS buffer at 37 °C for 40 min. Then the samples were washed with PBS three times. A mixture of 90% DMEM/F12 and 10% FBS was added to keep the samples wet during the examination period. According to the emission wavelength of CM-DiL, the filter was set to 570 LP and the laser was 553 nm in the fluorescence mode. In the reflection mode, the laser was 488 nm and the filter was set to 488/4 nm.

### Histology and Immunohistochemistry

The cell-seeded scaffold samples were taken out at week 2 and week 4 after culture, and then fixed with 10% neutral formalin, defatted with chloroform, demineralized with 10% ethylenedimine tetraacetic acid, and embedded in paraffin wax. The 5 μm thick sections were cut by microtom, stained with haematoxylin and eosin (H&E), Alcian blue, DAPI (Sigma, USA) and the immunohistochemical antibody of collagen II method, viewed and photographed under an optical or fluorescence microscope (Olympus microscope, Japan). H&E and DAPI were used to stain the ECM and cell nuclei. Alcian blue and immunohistochemical antibodies were used to stain glycosaminoglycan (GAG) and collagen II (C_II_), respectively.

It is commonly believed that the most specific ECM proteins of NP are GAG and C_II_. Therefore, the expression levels of protein GAG and C_II_ were used to evaluate whether rBMSCs differentiated towards NP-like cells.

### Gene quantification of NP cell markers by real-time qRT-PCR assay

The quantitative analysis of aggrecan and collagen II genes was adopted to evaluate the NP-like cells which differentiated from rBMSCs. RBMSCs were incubated in 48 well plates for 1, 2, and 4 weeks, after which the cell proliferation was determined by real-time qRT-PCR assays. Three samples were taken from each group for evaluation, and acellular scaffolds were used as the blank control group. The RNA of NP-like cells was extracted by a MiniBEST Universal RNA Extraction Kit (Takera, Japan). Real-time polymerase-chain reaction (PCR) was performed using SYBR^®^ Green PCR Master Mix (Takera, Japan), and glyceraldehyde-3-phosphate dehydrogenase (Invitrogen, USA) was used as the endogenous control. The RNA data were analyzed by ABI PRISM^®^ 7500 Sequence Detection System. Data were presented as mean with standard deviation.

### Statistics analysis

The quantitative data were presented as mean and standard deviation. The statistical significance of the differences between the experimental groups was evaluated by univariate ANOVA and LSD with the SPSS19.0. Value was *P* ≤ 0.05 was considered significant.

## Results and discussion

### Scaffold physicochemical characterization

Figure 1a, b showed the NP ECM-derived porous scaffolds produced into the regular cylinders shapes, those were 10 mm in diameter and 3 mm in height. They were spongy, porous, and white. Figure 1c, d, e was morphological and surface images of scaffold samples respectively, which indicated that the NP ECM-derived scaffolds still maintained the porous structure of the original ECM. The pore size of the scaffold was randomly selected from six fields under 1000× electron microscope, and six pore size were manually measured for each field. The SEM results showed that the pore sizes of the scaffolds range from 45 to 450 μm, very similar to the ideal size [33] required for tissue engineering. Meanwhile, the native NP tissue was very dense, and the porosity was less than 3 μm under ×5000 electron microscope. The porosity and water-binding capacity of the scaffolds were important features to evaluate the properties of biomaterials. Derived scaffolds with 81.28 ± 4.10% porosity were formed and these values were superior to those of the hexafluoroisopropanol-derived scaffolds prepared by salt leaching or gas forming [34]. The swelling ratio of NP-derived scaffolds prepared by freeze-drying method was 272.16 ± 33.30%, higher than that of collagen scaffolds, possibly due to the differences in hydrophilicity of the two types of proteins [35], but almost the same as that of polylactic acid scaffolds [36]. Regardless of the pore size of the scaffolds, water uptake of the aqueous-derived scaffolds in distilled water was above 93% within 24 h. The high water-binding capacity of the scaffolds could be attributed to the porous network structure.

### Cell proliferation in 3D scaffolds

#### MTT assay

**Fig. 2:**
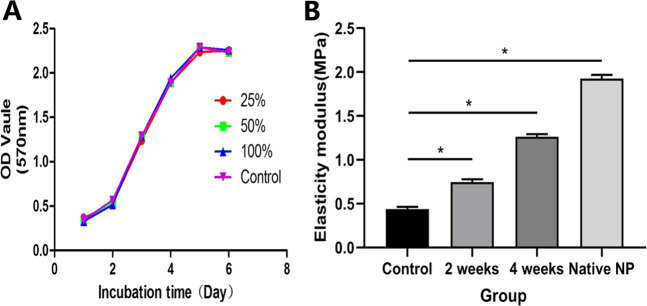
**a** Cytotoxicity of NP ECM-derived 3D porous scaffolds. RBMSCs were incubated with different concentrations of extract fluids obtained from scaffolds and complete medium as control group from 1 to 6 days. **b** Compressive elastic modulus of NP ECM-derived 3D porous scaffold and cell-seeded scaffolds cultured at week 2 and week 4. *P < 0.05. Data were mean ± SD, *n* = 6

As shown in Fig. 2a, MTT assay was adopted to assess the cell viability and proliferation of rBMSCs cultured in different 3D scaffolds for 1, 2, 3, 4, 5, and 6 days. As can be seen from the results of Univariate ANOVA and LSD summarized in Table 1, there was no significant difference between the four groups (*F* = 0.776, *P* = 0.702). There was also no significant difference in the proliferation of rBMSCs between the four groups (*P* = 0.233, 0.303, 0.094, 0.123, 0.064, and 0.262) at day 1, 2, 3, 4, 5, and 6 (Fig. 2a). In this study, the proliferation of the cells cultured in samples with different concentrations of 25, 50, and 100% was similar to that in blank control group. In Fig. 2a, the cell proliferation and growth showed best performance in scaffolds at day 5. The absorbance at 490 nm was almost 1.432, which was 3.87 times higher than that at day 1.

**Table 1:** The effect of different samples on MTT assay of rBMSCs for 1, 2, 3, 4, 5, and 6 days

Groups	Cultural time						Sum	*F*	*P*
	1 day	2 days	3 days	4 days	5 days	6 days			
25%									
$\overline x$	0.371	0.525	1.278	1.893	2.289	2.247	1.434	1.011	0.429
s	0.010	0.009	0.006	0.009	0.012	0.019	0.784		
50%									
$\overline x$	0.372	0.528	1.283	1.877	2.289	2.237	1.431	0.002	1.000
s	0.015	0.013	0.006	0.307	0.007	0.003	0.780		
100%									
$\overline x$	0.362	0.530	1.282	1.867	2.287	2.244	1.428	0.004	1.000
s	0.011	0.011	0.008	0.040	0.005	0.017	0.781		
Control									
$\overline x$	0.373	0.535	1.280	1.887	2.288	2.244	1.434	0.005	1.000
s	0.010	0.013	0.012	0.017	0.003	0.022	0.780		
Sum									
$\overline x$	0.370	0.530	1.281	1.881	2.290	2.243	1.432	1.149	0.332
s	0.012	0.011	0.008	0.027	0.007	0.016	0.773		
*F*	1.550	1.296	2.440	2.170	2.845	1.435	72528.027	(*F* = 0.776, *P* = 0.702)^b^	
*P*	0.233	0.303	0.094	0.123	0.064	0.262	0.000		

($$\overline x$$ ± s, OD value, *n* = 6)

^a^*F* statistic and *P* value of main effect

^b^*F* statistic and *P* value of crossover effect

#### Compressive elastic modulus

The mechanical properties of cell-seeded scaffolds were compared by means of a Zwick universal testing machine, After 2 and 4 weeks of incubation, respectively, the samples showed good mechanical effects except the control group (shown in Fig. 2b). The compressive elastic modulus of the scaffolds cultured for 4 weeks reached 0.12 MPa, which was significantly higher than that of the scaffolds cultured for 2 weeks (0.07 MPa) and that of the control group (0.04 MPa). Therefore, the compressive elastic moduli of the cell-seeded scaffolds increased with the proliferation of the NP cells and ECM in the scaffolds.

### Cell culture

#### Morphology of cell-seeded scaffold and cell proliferation

**Fig. 3:**
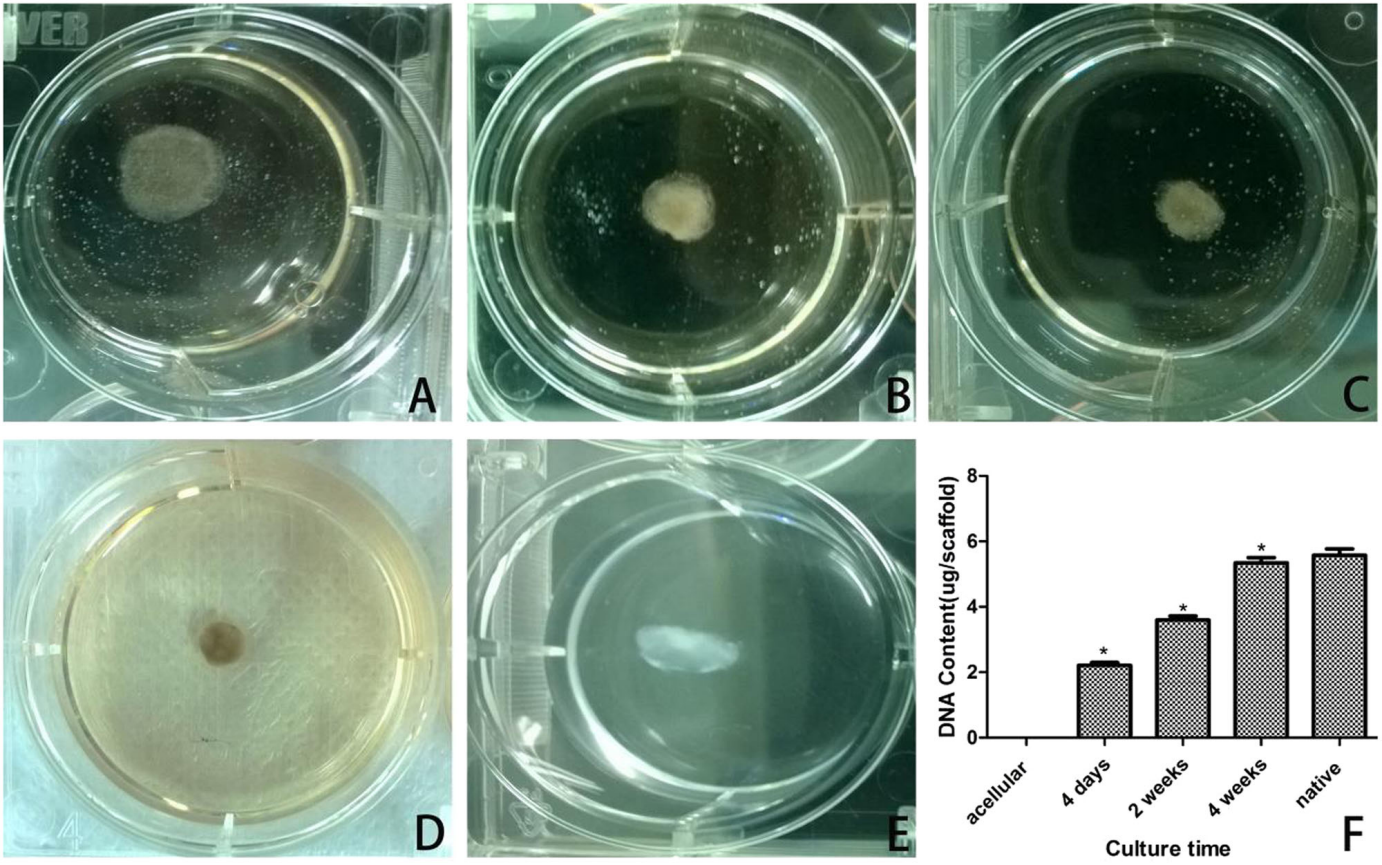
**a**–**e** Morphology changes of cell-seeded scaffold. **f** Total DNA contents at different culture time point. *P < 0.05. Data were mean ± SD, *n* = 6

NP cells were seeded on the three-dimensional scaffolds and cultured for 4 days, 2 and 4 weeks, respectively. Figure 3 showed the morphology of the cell-seeded scaffolds and total DNA content in the scaffolds observed by fluorescence spectrophotometer. The acellular scaffold group (Fig. 3a) was used as the negative control, and the normal NP (Fig. 3e) as the positive control. Morphology images of the cell-seeded scaffold (Fig. 3b–d) demonstrated that the cell-seeded scaffold was shrinking in shape, and gradually became dense at different time points. At week 4, the tenacity and flexibility of the cell-seeded scaffold was similar to those of the normal NP. This study suggested that this might be related to cell proliferation and ECM secretion. Most pores in the samples were filled with NP ECM. As a result, the NP microfilament was retracted by the newly secreted ECM. The increased DNA content in the scaffolds indicated that the samples in these groups (Fig. 3f) had good biocompatibility in 3D space. It showed that the number of NP cells at week 4 was larger than that at day 4 and week 2. Compared with the blank control group, the images implied that the proliferation of cells cultured in each scaffold increased with the prolongation of culture time, which was consistent with previous results of MTT assays.

#### Cell viability and attachment

**Fig. 4:**
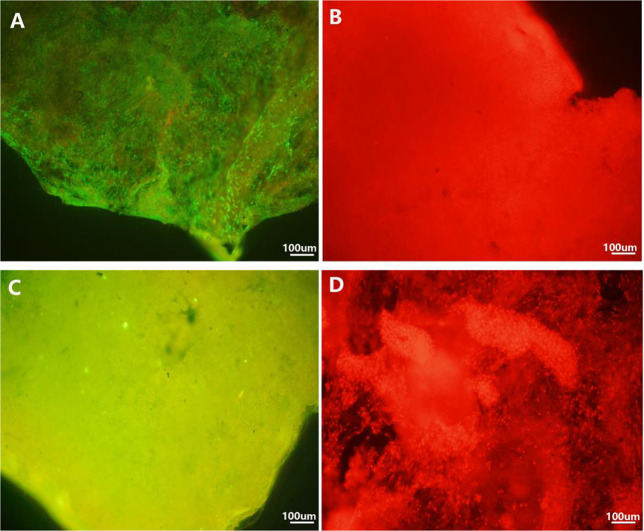
Fluorescence microscopy images of seeded cells with different staining in the scaffolds after 48 h of culture (×100): Green represented live cells which showed that seeded cells could survive in the scaffold

**Fig. 5:**
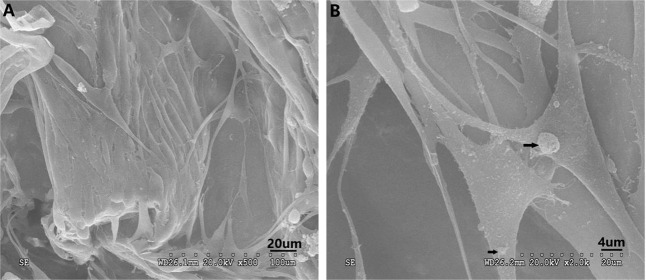
SEM micrographs of NP ECM-derived scaffold/induced BMSCs after 48 h of culturing. **a** rBMSCs covered most of the core and were distributed uniformly. **b** Matrix synthesis (indicated by the black arrows) around the cells

Figures 4 and 5 showed the cell viability and uniform distribution of rBMSCs which had been cultured for 2 days. These figures were observed by fluorescence microscope and SEM. Compared with the control groups (see Fig. 4c, d), fluorescence microscope images of rBMSCs (Fig. 4a) indicated that most of the live cells were stained green, and spread widely without any red lightspots (Fig. 4b). The number of rBMSCs attached to the scaffolds was indistinguishable, which implied that the cells were evenly distributed in the scaffold sample (Fig. 4a). Furthermore, the SEM pictures illustrated that the rBMSCs reached out “pseudopods” more obviously and progressively, polygonal in shape, fully spread (Fig. 5a, b). Meanwhile, the cells were surrounded by matrix synthesis and collagen fibers.

#### Cell labelling, DAPI staining, and cell tracking

**Fig. 6:**
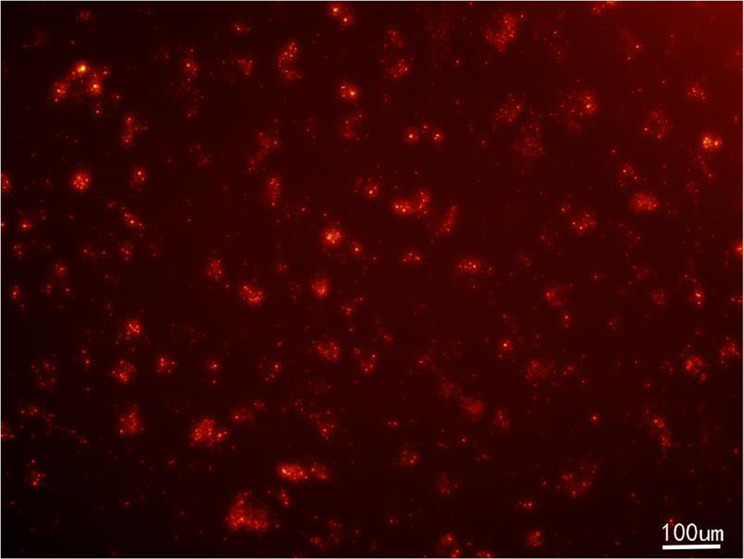
Fluorescence images of labeled rBMSCs in culture dish (×100)

**Fig. 7:**
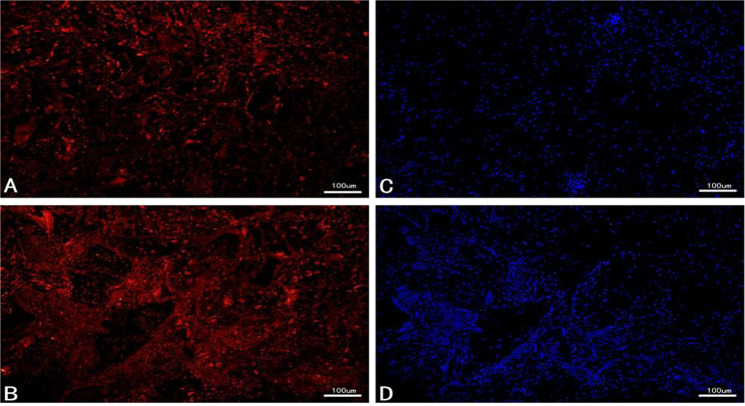
Fluorescent microscopy images of sections from cell-seeded scaffold: **a** CM-Dil labeled rBMSCs on scaffold at week 2 and **b** at week 4. **c** DAPI staining at week 2 implantation and **d** at week 4. The scale bars were 100 μm for all images

As shown in Fig. 6, rBMSCs labeled by the CM-DiL in the culture dish were stimulated with red fluorescence, indicating that the cell membranes of rBMSCs were labeled by the CM-DiL dye successfully. To observe the NP cells inside the scaffolds in more detail, experimenters cross-sectioned the complex. The specimen sections from groups of week 2 and week 4 exhibited strong red cell membrane fluorescence (Fig. 7a, b). Compared with that of week 2, the red fluorescence of week 4 was broader and more intense, indicating an increase in the number of nuclei at week 4. The blue nuclei stained with DAPI showed the same trend (Fig. 7c, d).

#### Histopathological and biochemical analysis

**Fig. 8:**
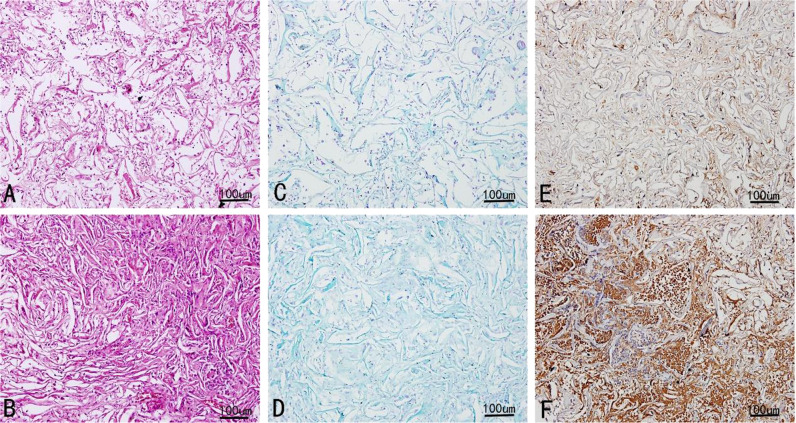
Histology and biochemistry analysis of complex: **a** H&E staining at week 2 and at week 4 (**b**). **c** Alcian blue staining at week 2 and at week 4 (**d**). **e** Collagen type II immunocytochemistry for 2 weeks and 4 weeks (**f**). As shown in **a** and **b**, respectively, a large number of cells distributed inside the pores of the scaffolds at the end of week 2 and week 4 in histopathological examinations. And a response in the scaffolds due to regeneration and a dense proteoglycans formation was observed with the help of alcian blue staining (**c**, **d**). Immunohistochemical staining was found to be positive for collagen II (**e**, **f**)

Histological and biochemical observations revealed the regeneration of rBMSCs, proteoglycans, and collagen II in the scaffolds within 4 weeks of incubation (see Fig. 8).

Figure 8a showed that the compact structure had not been fully formed at the end of the first 2 weeks, but there were abundant connective tissues in scaffold cavities observed by optical microscope. In spite of the regenerative reaction in the inner region, more immense proteoglycans and collagen II were formed (Fig. 8c, e). It showed that the 3D porous scaffolds exhibited the behavior of a typical bioactive composite, because of the prominence in new cell formation, the increase in cell activity, and diffusion of tissue similar to cartilage into the scaffold.

As shown in Fig. 8b, d, f, respectively, porous scaffolds at week 4 were further surrounded by active rBMSCs, proteoglycans, and collagen II formation areas. Figure 8b, d also demonstrated a large number of cells containing proteoglycans. The structure was more compact, and active collagen II formation occurred in some areas (Fig. 8f). Furthermore, the interface was more distinct because of the new collagen II formation. Meanwhile, significant differences were observed between the 2 and 4 week groups. The cells and ECM were nearly distributed across a single macropore and throughout the entire scaffold.

#### Gene quantification of NP cell markers by real-time qRT-PCR assay

**Fig. 9:**
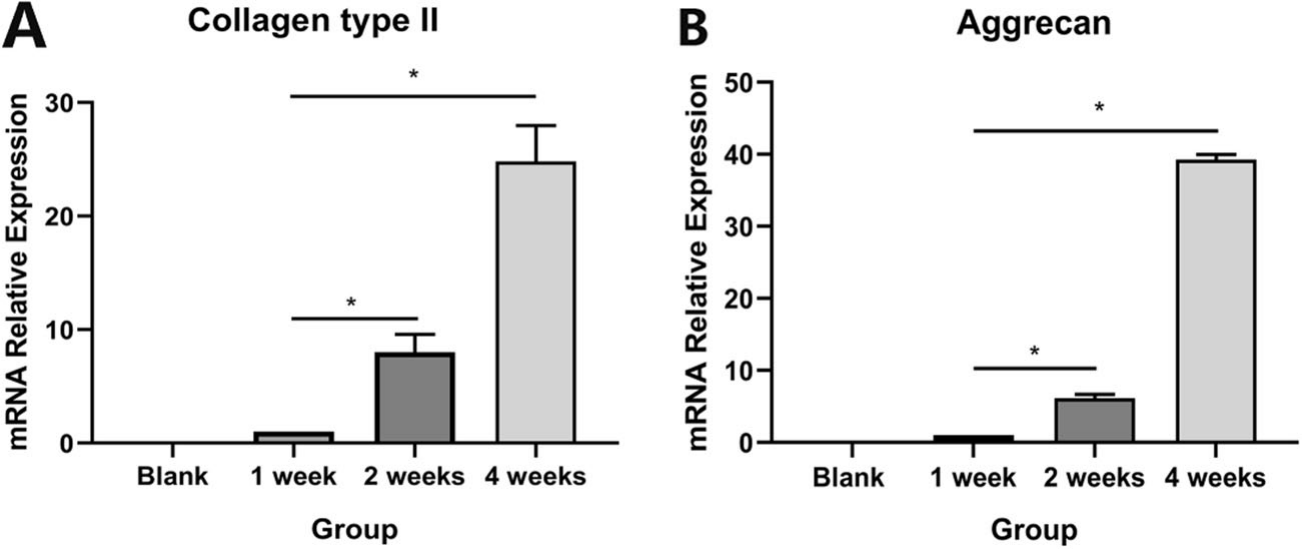
Quantitative analysis for **a** collagen type II and **b** Aggrecan

Figure 9 illustrated the total aggrecan and collagen II content of the scaffold samples cultured in vitro at different time points. It indicated that the contents of proteoglycans and type II collagen increased with the extension of the culture time. Meanwhile, these results showed similar tendencies. The proteoglycans and type II collagen contents at week 4 were significantly higher than those at week 2.

After comparing the morphology and immunohistological results of the scaffolds at different time after incubation, the experimenters found that the biomedical properties of scaffolds, especially the process of cell regeneration, were significantly different. The phenomena appearances of cell ingrowth, proteoglycans density increase, type II collagen formation in samples were very comparable with those at different time point, testifying the porous scaffolds had a good effect. The results revealed the good biocompatibility and conductive property of scaffolds.

## Conclusions

At present, there is still a lack of appropriate methods to treat early IDD with TE techniques. The key to the application of NP TE is to fabricate an ideal scaffold. This study suggested that the development of a novel NP ECM-derived 3D porous acellular scaffold might represent an appropriate and even successful solution. The 3D porous scaffold derived from rabbit NP tissue has good biocompatibility and loose porous structure. The rBMSCs survived well in vitro experiments, and the differentiation of rBMSCs could be promoted in the scaffold microenvironment. The elastic modulus of the cell-laden scaffold was close to that of normal NP in vitro. It was expected that the new scaffold with appropriate mechanical properties, larger pore sizes, and interconnected pores would be effective in treating IVD, as well as avoiding potential IDD and prolapsed disk.
